# Dynamic changes in peripheral blood lymphocyte trajectory predict the clinical outcomes of sepsis

**DOI:** 10.3389/fimmu.2025.1431066

**Published:** 2025-02-04

**Authors:** Dongkai Li, Jiahui Zhang, Wei Cheng, Guoyu Zhao, Xianli Lei, Yawen Xie, Na Cui, Hao Wang

**Affiliations:** ^1^ Department of Critical Care Medicine, Peking Union Medical College Hospital, Beijing, China; ^2^ Department of Critical Care Medicine, Beijing Jishuitan Hospital, Beijing, China

**Keywords:** sepsis, lymphocytes, trajectories, modeling, prognosis

## Abstract

**Background:**

Sepsis induces profound derangements in the immune system, including lymphopenia, which correlates with immunosuppression and poor prognosis. However, most evaluations of immunosuppression in sepsis patients rely on static, sporadic lymphocyte counts, lacking dynamic modeling over the disease course. This study aimed to apply latent class mixed modeling on longitudinal lymphocyte counts to uncover heterogeneous trajectory phenotypes in sepsis patients and assess their predictive value for clinical outcomes.

**Results:**

Four lymphocyte trajectory phenotypes were identified in the retrospective cohort (n=2,149) and externally validated (n=2,388): high–declining (α, 3.8%), stable–medium (β, 69.3%), high–increasing (γ, 3.2%), and stable–low (δ, 23.8%). The α phenotype exhibited the highest disease severity and mortality (25.9%) compared with other phenotypes in both cohorts. In the prospective cohort (n=1,056), all lymphocyte subset counts differed among phenotypes on admission (P <.001) and were lower in non-survivors (P<.05). Multivariable regression demonstrated that age, Acute Physiology and Chronic Health Evaluation-II score, heart rate, natural killer cell count, infection source, and lymphocyte trajectory phenotype were independent predictors of 28-day mortality. A nomogram combining these variables provided individualized risk estimations.

**Conclusions:**

The lymphocyte trajectories delineated novel dynamic phenotypes associated with divergent sepsis outcomes. Incorporating longitudinal trajectory modeling and lymphocyte subsets may improve prognostic risk assessment and guide the selection of immunotherapies tailored to specific immune phenotypes in sepsis patients.

**Clinical trial registration:**

https://www.chictr.org.cn/showproj.aspx?proj=18277, identifier ChiCTR-40 ROC-17010750.

## Background

Sepsis remains a major cause of mortality and morbidity in critically ill patients (1). Impaired immune function is integral to the pathogenesis of sepsis (2), and lymphopenia is correlated with immunosuppression and a poor prognosis (3–5). Lymphocyte apoptosis in lymphoid organs contributes to sepsis-induced lymphopenia (6); however, only sporadic and static lymphocyte counts are measured clinically. Dynamic fluctuations and kinetic patterns over the course of sepsis remain underexplored. Moreover, changes in lymphocyte subsets may better reflect immune derangements than total counts; however, few studies have examined their value for predicting sepsis outcomes (3). A deeper understanding of lymphocyte kinetics and derangements could enhance risk stratification and inform the selection of immunomodulatory therapy for patients with sepsis.

Latent class mixed modeling (LCMM) distinguishes heterogeneity in longitudinal data by identifying latent subgroups following distinct trajectory patterns (7). LCMM offers advantages over conventional growth modeling in that it accommodates individual variation in trajectories (8). Recent studies have used LCMM to model disease progression trajectories in Alzheimer’s disease (9), acute respiratory distress syndrome (10) and depression (11), revealing novel phenotypes. However, no studies have delineated the temporal lymphocyte phenotypes of patients with sepsis using LCMM.

Immunosuppression is increasingly recognized in the pathogenesis of sepsis, necessitating immunomodulatory therapy (12). Some trials have attempted to reverse lymphopenia and immune paralysis in patients with sepsis using antibodies against interleukin-7 (IL-7) and programmed cell death protein (PD)-1, as well as other agents, with mixed results (13, 14). Defining the immune trajectory phenotypes of patients with sepsis could better inform the selection of immunotherapies matched to specific immune states.

In this study, we used LCMM to unveil the heterogeneous peripheral blood lymphocyte trajectories of patients with sepsis and identify the correlations between these trajectories and patient outcomes. We further sought to externally validate the derived phenotypes and evaluate the associations between the lymphocyte counts on admission and the trajectory classification. Elucidating the kinetic patterns of lymphocyte subsets and identifying early derangements may provide insights into the prognosis and appropriate immunotherapies for patients with sepsis.

## Methods

### Study design and participants

**Figure 1:**
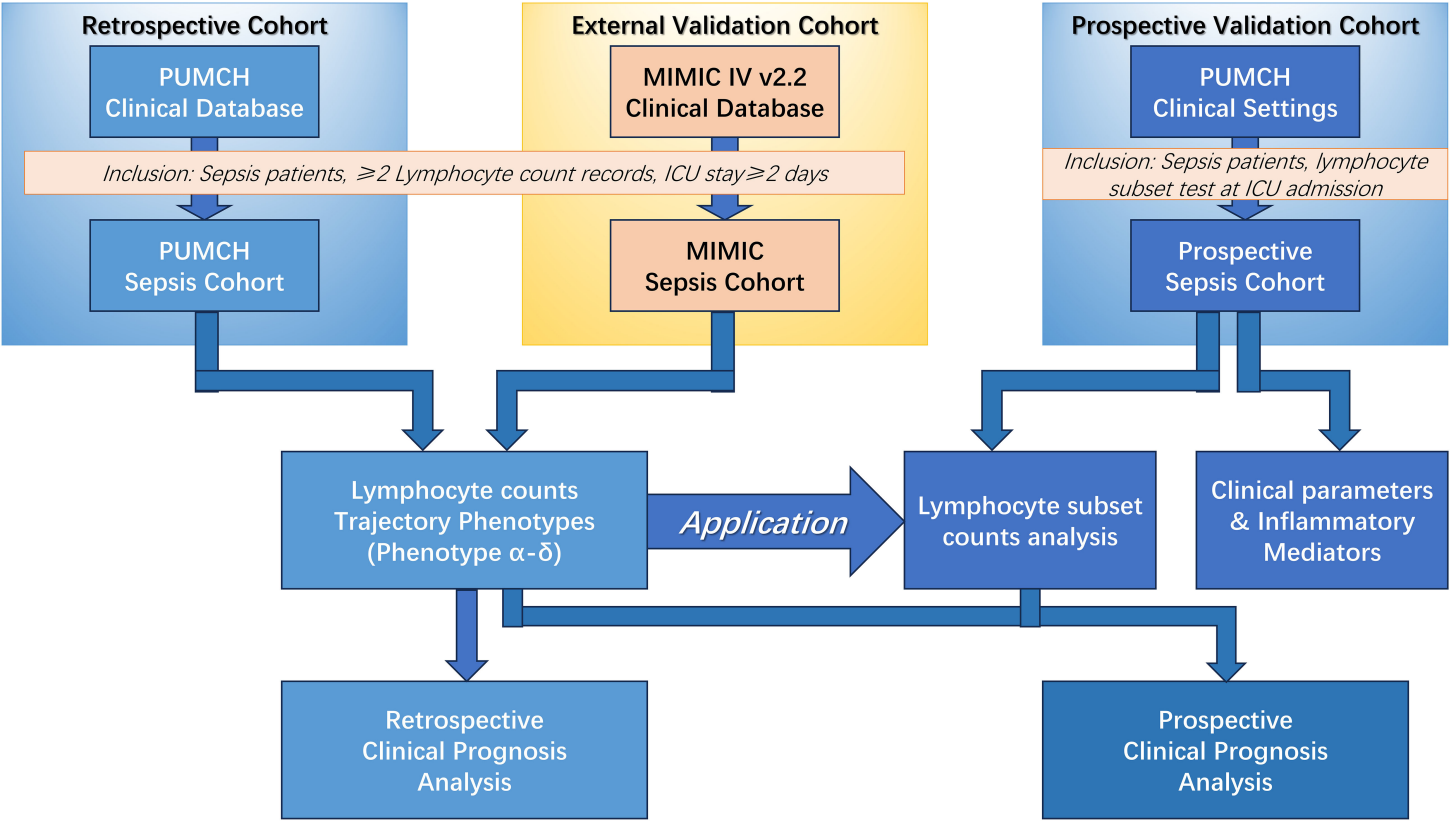
Schematic of the overall study. MIMIC, Medical Information Mart for Intensive Care; PUMCH, Peking Union Medical College Hospital; ICU, Intensive Care Unit.

This study included a retrospective cohort, an external validation cohort, and a prospective cohort (**Figure 1**). The retrospective cohort included adult patients (n = 2,149 patients) with sepsis who were admitted to the intensive care unit (ICU) of Peking Union Medical College Hospital, Beijing, China, between 2010 and 2019. Sepsis was defined according to the Sepsis-3 criteria (1). Patients were identified as having sepsis if they had a documented or suspected infection along with an acute increase of ≥2 points in the Sequential [Organ Failure Assessment] (SOFA) score. The inclusion criteria were (1) age ≥18 years; (2) ICU length of stay ≥48 hours; (3) no history of immunosuppression; and (4) at least two measurements of absolute peripheral lymphocyte count within the first 3 days of ICU admission. For external validation, data from the public Medical Information Mart for Intensive Care IV (MIMIC-IV v2.2) database were obtained according to the same inclusion criteria as the retrospective analysis cohort (n=2,388 patients). To explore the clinical significance of peripheral lymphocyte subset counts in the above phenotypes, a prospective analysis was conducted to evaluate quantitative changes in immune status along with their predictive value for 28-day mortality (n=1,056 patients). The sample sizes of retrospective cohort, external validation cohort and prospective validation cohort were determined by the available patients meeting inclusion criteria during the study periods. The prospective cohort was used for validation rather than *de novo* phenotype identification. Therefore, patients were classified into the previously identified phenotypes using the established criteria from the retrospective analysis. This approach was chosen to validate the reproducibility of the phenotype classifications. This study was approved by the institutional review board of Peking Union Medical College Hospital (approval number JS-2800 and K3148). This study was conducted in accordance with the ethical standards of the Declaration of Helsinki (as revised in 2013). Informed consent was obtained from all patients, and the study is registered at chictr.org.cn (identifier ChiCTR-ROC-17010750).

### LCMM analysis

LCMM is a type of finite mixture model that can identify unobserved heterogeneity in longitudinal data by classifying individuals into distinct latent trajectory classes (9). LCMM assumes that the population comprises a mixture of distinct subgroups characterized by different patterns of evolution over time. It uses a polynomial regression model to approximate the shape of the trajectory within each latent class. Maximum-likelihood estimation is applied to determine the optimal number of latent classes that best fit the data based on certain criteria, such as the Bayesian information criterion (BIC) (8). Compared with conventional growth modeling, LCMM better accounts for individual variability in trajectories by allowing subject-specific random effects within each class (7). Recent studies have used LCMM to uncover novel phenotypes of disease progression for certain conditions (9–11). In this study, lymphocyte counts were available at four time points (0, 24, 48, and 72 hours after ICU admission) for each patient and were used for trajectory modeling. Then the LCMM analysis was performed using the lcmm package of R software, which contains tools for fitting LCMM and deriving trajectories (8). LCMM was applied to the retrospective cohort to identify distinct lymphocyte trajectory phenotypes over the course of sepsis. The optimal number of clusters was determined using Bayesian information criterion (BIC) scores. These derived phenotype patterns were then used to classify patients in both the external validation and prospective cohorts.

### External validation cohort

For external validation, we used data from the MIMIC-IV v2.2 database, which is a publicly available database (12). MIMIC-IV contains deidentified health data associated with over 200000 ICU admissions between 2008 and 2019 at Beth Israel Deaconess Medical Center in Boston, MA, USA (12). The database includes data on vital signs, medications, diagnostic codes, laboratory values, imaging reports, clinical notes, and mortality. For the validation cohort, MIMIC-IV was queried using Structured Query Language to identify patients who met the same inclusion criteria as the derivation (retrospective) cohort.

### Clinical data collection and outcomes

Demographics, clinical data, and infection source details were extracted from the hospital electronic medical records system and MIMIC-IV database. In all of the retrospective, external validation and prospective validation analyses, patient demographics; clinical data, such as mean arterial pressure, heart rate, duration of ventilator treatment, Acute Physiology and Chronic Health Evaluation (APACHE)-II score (or Simplified Acute Physiology Score (SAPS) for the MIMIC-IV external validation cohort), and Sequential Organ Failure Assessment (SOFA) score; and outcomes, such as the duration of ICU and in-hospital stay and 28-day mortality, were recorded.

In the prospective analysis, blood samples were obtained upon ICU admission and for 3 consecutive days after ICU admission, including complete blood counts; C-reactive protein and procalcitonin concentrations; blood gas analysis. Upon admission to ICU, the immunological assessment was conducted on peripheral blood specimens at PUMCH laboratory facilities, following previously established protocols (13). The procedure involved taking fresh whole blood samples treated with EDTA anticoagulant, which were then stained using fluorochrome-conjugated monoclonal antibodies. These antibodies targeted specific combinations: CD3/CD8/CD4, CD3/CD16CD56/CD19, along with appropriate isotype controls (sourced from Immunotech, France). The samples underwent three-color flow cytometric analysis using an EPICS-XL flow cytometer (Beckman Coulter, Brea, CA, USA) to identify and quantify T-lymphocytes (CD3+), their CD4+ and CD8+ subpopulations, B-lymphocytes (CD19+), and natural killer cells (CD3-CD16+CD56+). The specific fluorescent monoclonal antibodies utilized included CD45-FITC/CD4-RD1/CD8-ECD/CD3-PC5, CD45-FITC/CD56-RD1/CD19-ECD/CD3-PC5, and CD16-PE (all from Beckman Coulter, Brea, CA, USA). Additionally, serum immunoglobulin levels (IgA, IgG, and IgM) and complement components (C3 and C4) were measured using rate nephelometry on an Array 360 system (Beckman Coulter, Brea, CA, USA). Measurements of immunological parameters and lymphocyte subset counts were performed using peripheral blood samples obtained at Peking Union Medical College Hospital.

### Statistical analysis

Clinical characteristics and outcomes were compared among the lymphocyte trajectory phenotypes by analysis of variance or the Kruskal–Wallis test. Continuous variables are expressed as the mean ± standard deviation or median (interquartile range), as appropriate. The Student’s t-test or the Mann–Whitney U test was used for comparisons. Categorical variables are expressed as frequencies and proportions and were compared using the χ^2^ test or Fisher’s exact test, as appropriate. Survival analyses were performed using the Kaplan–Meier method with the log-rank test. Multivariable logistic regression was used to identify independent predictors of 28-day mortality for nomogram construction. All statistical analyses were performed using R statistical software (version 4.3.0, The R Foundation for Statistical Computing, https://www.r-project.org/) and RStudio (2023.06.2 Build 561, RStudio, Posit Software, PBC, http://www.rstudio.com/) with the *lcmm*, *survminer*, and *survival* packages. A nomogram based on the selected final model was constructed from the prospective cohort using the rms package. A significance level of *p* < 0.05 from two-sided statistical tests (e.g. t-test, ANOVA) was used to determine statistical significance for all analyses.

## Results

### Retrospective cohort trajectory analysis

The retrospective cohort included 2149 patients with sepsis who were admitted to the ICU (**Supplementary Figure S1**). The mean age of the patients was 59.39 ± 17.58 years, and 1,310 of 2,149 (61.0%) were male. The mean APACHE-II score was 19.7 ± 7.7, and the mean SOFA score was 9.1 ± 4.0. Overall, 1220 of 2149 patients (56.8%) had pulmonary infection as the main infection source, and the overall 28-day mortality rate was 16.3% (351/2149) (**Supplementary Table S1**).

**Figure 2:**
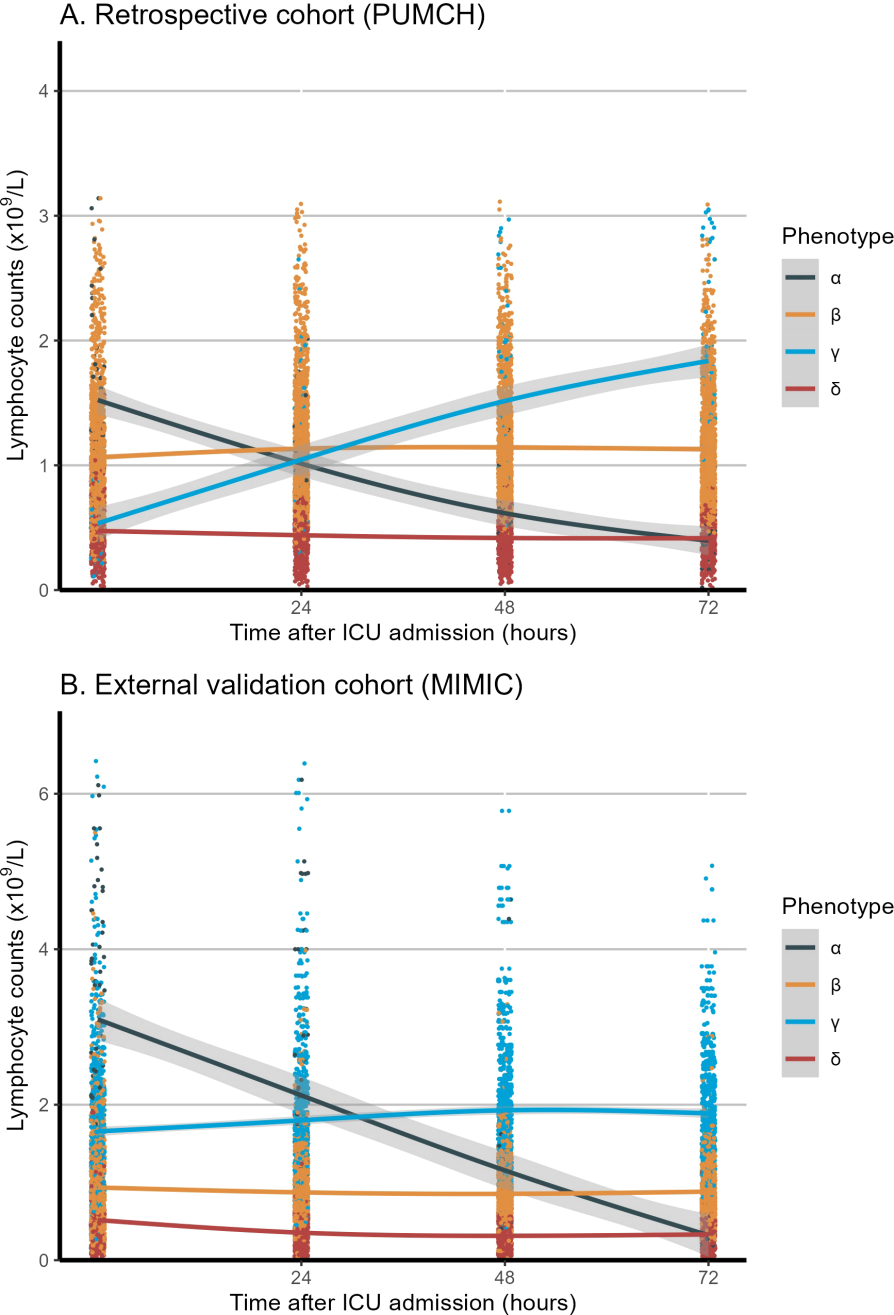
Longitudinal lymphocyte count trajectories in the first 72 hours among the four phenotypes. **(A)** Retrospective cohort (PUMCH). **(B)** External validation cohort (MIMIC). The x-axis represents the timing of lymphocyte measurement (0, 24, 48, and 72 hours after ICU admission), and the y-axis represents the lymphocyte counts (x10^9^/L). The shaded area represents the confidence interval around the smooth curve, with a confidence level of 0.95. MIMIC, Medical Information Mart for Intensive Care; PUMCH, Peking Union Medical College Hospital; ICU, Intensive Care Unit.

The LCMM analysis of longitudinal peripheral lymphocyte counts revealed four phenotypes with heterogeneous trajectories (**Figure 2**): high–declining (α; n = 81), stable–medium (β; n = 1,488), high–increasing (γ; n = 69), and stable–low (δ; n = 511). The optimal four clusters were determined by the BIC–cluster curve (**Supplementary Figure S2**). Based on these identified patterns, classification thresholds were established with α phenotype showing Initial count >1.5×10^9^/L with negative slope, β phenotype maintaining 0.8-1.5×10^9^/L with minimal variation, γ phenotype with initial count >1.5×10^9^/L with positive slope, and δ phenotype maintaining counts <0.8×10^9^/L with minimal variation. These phenotypes and their classification criteria were subsequently validated in the MIMIC-IV cohort (n=2,388) and applied to classify patients in the prospective cohort (n=1,056).

Compared with the other phenotypes, patients classified as the α phenotype were older and had higher APACHE-II and SOFA scores (suggesting greater disease severity), a higher lactate concentration, lower oxygenation, longer durations of vasopressor and mechanical ventilation use, and higher 28-day mortality (25.9% vs. 14.0%–16.3% for the other phenotypes, *p* < 0.001). The γ phenotype exhibited the lowest disease severity and the lowest mortality rate. There were no differences in sex, heart rate, temperature, respiratory rate, or source of infection among the groups.

### External validation cohort

The external MIMIC-IV cohort included 2388 patients with sepsis who were admitted to the ICU (**Supplementary Figure S1**). The mean age of the patients was 64.76 ± 16.21 years, and 1059 of 2388 patients (44.3%) were male. The mean SAPS II score was 43.75 ± 14.66, and the mean SOFA score was 4.20 ± 2.36, reflecting moderate-to-severe disease severity. In the external validation cohort, the overall 28-day mortality rate was 25.7% (613/2388) (**Supplementary Table S2**).

**Figure 3:**
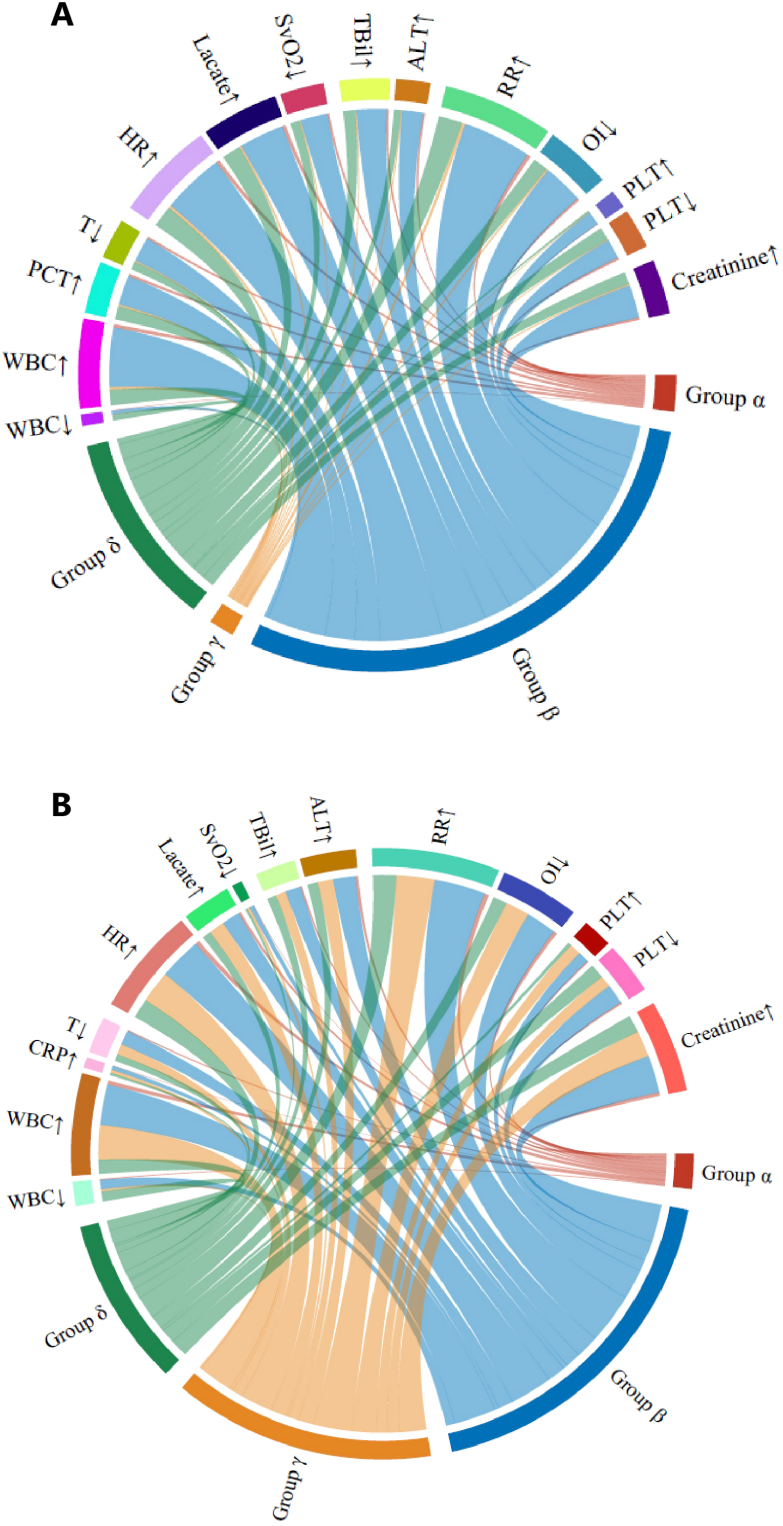
Chord diagrams showing the relationship among the four lymphocyte trajectory phenotypes and abnormal clinical variables. **(A)** Retrospective (PUMCH) cohort. **(B)** External validation (MIMIC) cohort. In each diagram, the ribbons connect from an individual phenotype to an indicator if the cohort with that phenotype included patients in which the indicator was greater or less than the normal range. ALT, alanine transaminase; AST, aspartate transaminase; CRP, C-reactive protein; ESR, erythrocyte sedimentation rate; GCS, Glasgow Coma Scale; HR, heart rate; INR, international normalized ratio; OI, oxygenation index; PaO_2_, partial pressure of oxygen; PLT, platelet; RR, respiration rate; SBP, systolic blood pressure; SvO_2_, venous oxygen saturation; T, temperature; TBil, total bilirubin; WBC, white blood cell.

The LCMM trajectory analysis confirmed similar longitudinal peripheral lymphocyte patterns (**Figure 2**) for the α (n = 91), β (n = 1,043), γ (n = 780), and δ (n = 474) phenotypes. The optimal four clusters were determined by the BIC–cluster curve (**Supplementary Figure S2**). Similar to the retrospective cohort, patients classified as the α phenotype were more severely ill, with a higher lactate concentration, longer vasopressor and ventilation use, and a higher 28-day mortality rate (38.5% vs. 21%–32% for the other phenotypes, *p* < 0.001). The γ phenotype continued to display the lowest disease severity and mortality risk, while the α phenotype showed the highest disease severity and mortality risk. Heart rate, temperature, and respiratory rate did not differ among the phenotypes. In the survival analysis of both the retrospective (PUMCH) and external validation (MIMIC) cohort, the α phenotype showed significantly lower survival over the 28-day period compared to the other three phenotypes (*p* < 0.001), as showed in **Supplementary Figure S3**. The dynamic changes in SOFA scores and subsystem scores over time are shown in **Supplementary Figure S4** for the retrospective and validation cohorts. The progression of organ dysfunction aligned with the clinical deterioration in the more severe α phenotype compared with the less severe γ phenotype. For both the restrospective and external validation cohorts, the chord diagrams in **Figure 3** demonstrate the relationship between the trajectory phenotypes and abnormalities in clinical variables, conveying the associations between immune trajectories and organ dysfunction.

### Prospective cohort and nomogram model construction

The prospective cohort included 1056 patients who were admitted to the ICU of Peking Union Medical College Hospital (**Supplementary Figure S1**). The mean age of the patients was 60.0 ± 16.6 years, and 688 of 1056 patients (65.2%) were male. The mean APACHE-II score was 19.8 ± 7.5, and the mean SOFA score was 6.9 ± 3.0. Overall, 753 patients (71.3%) had pulmonary infection as the main infection source, and the overall 28-day mortality rate was 16.4% (173/1056) (**Supplementary Tables S3**, **S4**).

Patients were classified into the four phenotypes: α (n = 241), β (n = 535), γ (n = 225), and δ (n = 55). Patients classified as the α phenotype were older and had higher APACHE-II scores, a longer ICU stay, and higher 28-day mortality (22.8% vs. 11.6%–15.6% for the other phenotypes, *p* < 0.001). The sex distribution was similar among phenotypes. All lymphocyte subset counts measured upon admission differed significantly among the phenotypes and were lowest in the δ group (**Supplementary Figure S5**). The lymphocyte subset counts were also lower among non-survivors than among survivors (**Supplementary Table S4**).

**Figure 4:**
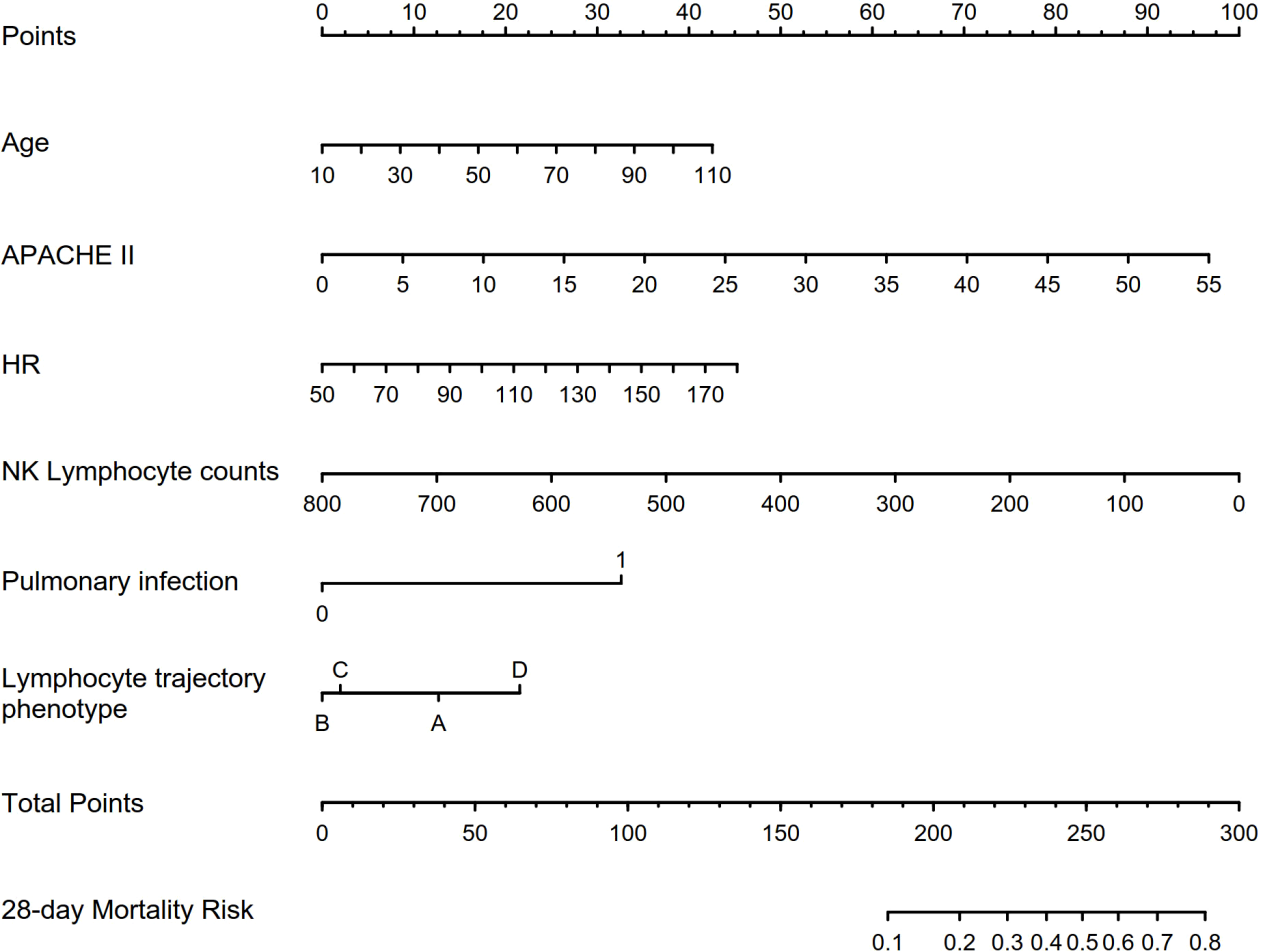
Characteristics included in the nomogram to predict 28-day mortality in patients with sepsis. APACHE-II, Acute Physiology and Chronic Health Evaluation II; HR, heart rate; NK, natural killer cell.

Binary logistic regression was conducted to identify independent predictors of 28-day mortality in the prospective cohort, as showed in **Table 1**. Univariable logistic regression first examined the association between each variable and mortality. Variables with *p* values of <0.1 were entered into the multivariable logistic regression model using the forward stepwise method. The variables of age, APACHE-II score, heart rate, NK cell count, infection source (pulmonary or non-pulmonary), and lymphocyte trajectory phenotype were independent predictors of 28-day mortality (*p* < 0.05). A nomogram prediction model was constructed using these four independent predictors of 28-day mortality (**Figure 4**). The nomogram developed in this study provides a visual representation of the independent predictors of 28-day mortality, allowing clinicians to estimate individualized risk based on a patient’s age, APACHE-II score, heart rate, NK cell count, infection source, and lymphocyte trajectory phenotype. This nomogram can aid in risk stratification and triage decisions for sepsis patients upon ICU admission.

**Table 1:** Multivariable logistic regression analysis of predictors of 28-day mortality in the prospective cohort.

Variables	Unadjusted		Adjusted	
	OR (95% CI)	*p* value	OR (95% CI)	*p* value
Age	1.016 (1.005, 1.026)	<.005	**1.013 (1.000, 1.026)**	**.059**
APACHE-II	1.086 (1.062, 1.109)	<.005	**1.033 (1.059, 1.086)**	**<.005**
HR	1.019 (1.010, 1.027)	<.005	**1.010 (1.000, 1.019)**	**.040**
T	1.360 (1.150, 1.607)	<.005	1.170 (0.962, 1.423)	.117
RR	1.013 (0.991, 1.035)	.246	1.004 (0.981, 1.028)	.724
OI	1.000 (0.999, 1.001)	.892	1.001 (1.000, 1.001)	.262
BLC	0.999 (0.997, 1.001)	.154	0.998 (0.999, 1.001)	.462
TLC	0.999 (0.999, 1.000)	.001	0.992 (0.984, 1.001)	.088
CD4+ TLC	0.999 (0.998, 1.000)	.065	1.008 (0.999, 1.017)	.074
CD8+ TLC	0.997 (0.996, 0.999)	<.005	1.007 (0.998, 1.016)	.126
CD4/CD8	1.098 (1.026, 1.176)	.007	1.013 (0.916, 1.121)	.797
NK LC	0.992 (0.989, 0.995)	<.005	**0.994 (0.997, 1.000)**	**.051**
Infection source*	3.152 (1.886, 5.267)	<.005	**2.890 (1.655, 5.048)**	**<.005**
Lymphocyte trajectory phenotype				
α	0.479 (0.257, 0.891)	.020	**0.271 (0.559, 1.154)**	**.116**
β	0.212 (0.116, 0.389)	<.005	**0.179 (0.403, 0.910)**	**.029**
γ	0.298 (0.155, 0.573)	<.005	**0.384 (0.181, 0.815)**	**.013**
δ	1.000	—	**1.000**	**—**

*Compared between the patients with pulmonary infection vs. non-pulmonary.

OR, odds ratio; CI, confidence interval; APACHE-II, Acute Physiology and Chronic Health Evaluation II; HR, heart rate; T, temperature; RR, respiratory rate; OI, oxygenation index; BLC, B-lymphocyte count; TLC, T-lymphocyte count; NK LC, natural killer lymphocyte count; CNS, central nervous system; SST, skin, and soft tissue; UTI, urinary tract infection.

P values <0.05 were displayed in bold and considered as independent predictors of 28-day mortality.

## Discussion

In this study, we identified four peripheral blood lymphocyte trajectory phenotypes in patients with sepsis and determined their associations with clinical outcomes. Few studies have delineated heterogeneity in longitudinal lymphocyte kinetics in patients with sepsis. To our knowledge, this is the first study to use LCMM to identify clinically meaningful temporal phenotypes in patients with sepsis. The lymphocyte trajectories likely reflect sequential immune states in sepsis progression (14). The α phenotype represented an initial proinflammatory phase followed by immunosuppression, while the γ phenotype denoted delayed immune activation. Identifying these phenotypes and understanding their correlations with prognosis in patients with sepsis are significant innovations.

Several previous studies have attempted to identify sepsis phenotypes using clustering methods on admission clinical and biomarker data (15). These approaches defined phenotypes based on inflammation phenotypes, metabolic phenotypes, and static immuno-biological phenotypes (14, 16, 17). However, prior works were limited by solely utilizing cross-sectional data at discrete timepoints, often just at ICU admission. The temporal dynamics and heterogeneity of sepsis progression over time were not well delineated. No study has characterized potential heterogeneous trajectories of immune cells or biomarkers. Our analysis addresses this gap through modeling longitudinal lymphocyte counts to uncover kinetic phenotypes over the early sepsis course. The trajectories provide novel insights beyond conventional static clusters and reflect potential sequential immune states in sepsis immunopathology.

We also demonstrated that quantification of lymphocyte subsets on admission allowed early risk stratification. Previous small-sample studies have delineated lymphocyte trajectories in patients with sepsis, but these studies were limited by their small sample sizes and lack of external validation (3, 5). The present study leveraged LCMM in a much larger cohort and validated the findings externally, better characterizing lymphocyte trajectories, which likely reflect sequential immune states (14). We also expanded the analysis across multiple lymphocyte subsets and evaluated their significance combining with phenotypes. Incorporating lymphocyte subset variables with conventional risk factors improved mortality prediction, as evidenced by the nomogram. This study uniquely analyzed lymphocyte trajectories and lymphocyte subsets at ICU admission to improve the risk assessment of patients with sepsis. Interestingly, quantifying NK cells enabled additional mortality prediction, affirming the role of NK cells in immunity. NK cell dysfunction occurs in sepsis, contributing to immunosuppression (18). Low NK cell counts at sepsis onset are associated with secondary infections and mortality (19). Therefore, evaluating NK cells at admission may identify high-risk patients requiring intervention.

The lymphocyte trajectory phenotypes identified in this study provide perspectives into the longitudinal immune dysregulation in sepsis (11). The α phenotype (high–decline) and δ phenotype (stable–low) manifest immunosuppressive states, supporting the concept of sepsis-induced immunoparalysis (10, 20). The γ phenotype (high–increase) and β phenotype (stable–medium) present contrasting activating and balanced immunity. These trajectories may indicate the significance of staging sepsis into sequential immune phenotypes. Further research should investigate whether transitioning patients’ trajectories to more favorable states may improve outcomes. Some studies have trialed immunostimulatory therapies, such as anti-IL-7 and anti-PD-1 antibodies, in patients with sepsis to counter immunosuppression, with mixed results (21, 22). Our findings provide a framework to select immunotherapies matched to a patient’s immune trajectory phenotype, which could optimize the treatment response.

This study had several limitations. First, this was a single-center retrospective study with inherent selection bias. The lymphocyte trajectory phenotypes require further external validation in other cohorts and settings. Second, the effects of medications, fluids, and interventions on lymphocyte counts were not assessed. Third, while our prospective cohort included detailed lymphocyte subset data, we focused primarily on cell counts rather than functional analyses. Future studies should incorporate functional assays of NK cells and other lymphocyte subsets to better understand their mechanistic roles in different phenotypes. Additionally, serial measurements of lymphocyte subsets could provide insights into the dynamic changes in immune cell populations and reveal additional phenotype-specific patterns. While this study identified associations between lymphocyte trajectory phenotypes and clinical outcomes, it did not establish a causal relationship. Further studies should focus on prospectively validating the lymphocyte trajectory phenotypes, delineating the potential mechanisms of the phenotypes, and explore whether interventions that modulate lymphocyte kinetics can alter the trajectory phenotypes and improve outcomes.

Another potential limitation of this study is the heterogeneity in the sepsis cohorts and the unknown stage of sepsis at the time of ICU admission. The lymphocyte trajectory phenotypes identified may reflect different stages of the same underlying sepsis process, combined with variable disease severity. For example, the high-declining phenotype could represent early, actively propagating sepsis, while the stable phenotypes may reflect later, more stable stages. The increasing phenotype might indicate recovering patients. Also, while our study demonstrates the utility of longitudinal lymphocyte trajectory analysis in sepsis, the need for 72-hour data collection may delay phenotype classification and clinical application. Future studies incorporating longitudinal lymphocyte subset measurements could provide deeper insights into immune dysfunction patterns and the relationship between lymphocyte kinetics, sepsis stage, and outcomes.

Despite its limitations, this study, which identified dynamic lymphocyte trajectories, provides novel insights into the heterogeneous immune pathophysiology of patients with sepsis over time. Early quantification of changes in lymphocyte counts and classification of these changes into phenotypes predicts clinical outcomes. Incorporating this approach could enhance prognostic risk stratification and guide the selection of immunotherapies tailored to distinct immune phenotypes in patients with sepsis.

## Conclusions

Patients with sepsis demonstrate heterogeneous temporal peripheral blood lymphocyte trajectory phenotypes that correlate with disease severity and mortality risk. LCMM of dynamic lymphocyte counts facilitates the classification of patients with sepsis into phenotypes based on their lymphocyte trajectories, with divergent clinical outcomes. Quantifying lymphocyte subsets at ICU admission serves as an early prognostic tool for mortality prediction. Incorporating the analysis of lymphocyte counts, both longitudinally and at admission, could enhance risk stratification and guide the selection of immunotherapy strategies for patients with sepsis.

## Data Availability

The raw data supporting the conclusions of this article will be made available by the authors, without undue reservation.
