# Supplementary material for: Dynamic changes in peripheral blood lymphocyte trajectory predict the clinical outcomes of sepsis
Source: Front Immunol. 2025 Feb 4;16:1431066. doi: 10.3389/fimmu.2025.1431066 (PMC11832464; doi:10.3389/fimmu.2025.1431066)
Supplement: Supplementary file 1 [file DataSheet1.pdf]

Supplemental Materials

Table S1. Comparison of clinical characteristics among the phenotypes of patients in the retrospective cohort.

Characteristic	Overall	α	β	γ	δ	*p*
**n**	2149	81	1488	69	511	
**Age (mean (SD))**	59.39 (17.58)	59.56 (16.18)	59.23 (17.58)	54.70 (20.16)	60.46 (17.36)	0.073
**Sex (Male, %)**	1310 (61.0)	42 (51.9)	929 (62.4)	40 (58.0)	299 (58.5)	0.126
**APACHE-II (mean (SD))**	19.66 (7.67)	23.03 (8.00)	19.10 (7.48)	17.08 (7.01)	21.05 (7.89)	<0.001
**SOFA (mean (SD))**	9.06 (4.01)	11.11 (3.79)	8.67 (3.98)	8.22 (3.22)	9.98 (3.99)	<0.001
**LY-D1 ($x10^9$/L, mean (SD))**	0.91 (0.53)	1.44 (0.52)	1.06 (0.50)	0.51 (0.27)	0.45 (0.22)	<0.001
**LY-D2 ($x10^9$/L, mean (SD))**	0.97 (0.53)	0.99 (0.44)	1.15 (0.50)	0.99 (0.49)	0.43 (0.18)	<0.001
**LY-D3 ($x10^9$/L, mean (SD))**	0.96 (0.53)	0.61 (0.24)	1.15 (0.46)	1.44 (0.63)	0.41 (0.16)	<0.001
**LY-D4 ($x10^9$/L, mean (SD))**	0.95 (0.52)	0.45 (0.18)	1.13 (0.44)	1.78 (0.64)	0.42 (0.16)	<0.001
**HR (bpm, mean (SD))**	101.94 (21.69)	105.80 (24.75)	101.43 (21.38)	103.26 (22.18)	102.64 (21.99)	0.245
**T (℃, mean (SD))**	36.85 (1.08)	37.19 (1.29)	36.87 (1.08)	37.00 (1.06)	36.71 (1.04)	<0.001
**RR (/min, mean (SD))**	20.94 (6.72)	22.14 (7.26)	20.75 (6.49)	21.19 (6.69)	21.27 (7.27)	0.171
**OI (mmHg, median [IQR])**	215.00 [153.55, 293.30]	205.70 [149.50, 285.48]	219.55 [159.35, 300.00]	227.50 [184.55, 294.35]	204.25 [135.22, 280.53]	<0.001
**Lactate (mmol/L, median [IQR])**	2.10 [1.30, 3.80]	3.30 [1.80, 6.32]	2.00 [1.30, 3.50]	2.00 [1.37, 3.25]	2.40 [1.40, 4.00]	<0.001
**Length of ICU stay (days, median [IQR])**	7.00 [3.90, 13.80]	7.80 [4.30, 13.80]	7.10 [3.90, 13.90]	5.50 [3.00, 8.90]	7.00 [3.90, 13.90]	0.011
**Duration of vasopressor (hours, median [IQR])**	106.22 [50.00, 220.00]	101.50 [58.42, 241.04]	106.10 [51.00, 214.50]	75.06 [39.19, 131.81]	108.50 [49.30, 242.00]	0.351
**Duration of mechanical ventilation (hours, median [IQR])**	111.10 [47.75, 239.17]	156.67 [63.07, 301.25]	109.28 [42.00, 221.30]	64.67 [35.18, 127.57]	123.00 [55.00, 276.25]	0.001
**Infection source (%)**						
**CNS**	18 (0.8)	0 (0.0)	14 (0.9)	1 (1.4)	3 (0.6)	<0.001

Characteristic	Overall	α	β	γ	δ	*p*
**SST**	79 (3.7)	0 (0.0)	63 (4.2)	6 (8.7)	10 (2.0)	
**Valves**	84 (3.9)	6 (7.4)	68 (4.6)	5 (7.2)	5 (1.0)	
**Lung**	1220 (56.8)	50 (61.7)	835 (56.1)	31 (44.9)	304 (59.5)	
**Intra-abdominal**	502 (23.4)	18 (22.2)	328 (22.0)	15 (21.7)	141 (27.6)	
**UTI**	10 (0.5)	0 (0.0)	7 (0.5)	3 (4.3)	0 (0.0)	
**Others**	140 (6.5)	5 (6.2)	104 (7.0)	5 (7.2)	26 (5.1)	
**Pleural**	53 (2.5)	1 (1.2)	39 (2.6)	1 (1.4)	12 (2.3)	
**Bloodstream**	42 (2.0)	1 (1.2)	30 (2.0)	2 (2.9)	9 (1.8)	
**Mediastinum**	1 (0.0)	0 (0.0)	0 (0.0)	0 (0.0)	1 (0.2)	
**28-Day Mortality (%)**	351 (16.3)	21 (25.9)	209 (14.0)	5 (7.2)	116 (22.7)	<0.001

Abbreviations: SD, standard deviation, IQR, interquartile range, APACHE-II, Acute Physiology and Chronic Health Evaluation II; SOFA, Sequential Organ Failure Assessment; HR, heart rate; T, temperature; RR, respiratory rate; OI, oxygenation index; LY, lymphocyte count; D1–D4, 1–4 days after intensive care unit admission. CNS, central nervous system; SST, skin, and soft tissue; UTI, urinary tract infection.

Table S2. Comparison of clinical characteristics among the phenotypes of patients in the external validation cohort.

Characteristic	Overall	α	β	γ	δ	*p*
**n**	2388	91	1043	780	474	
**Age (mean (SD))**	64.76 (16.21)	62.52 (17.34)	65.89 (15.83)	63.05 (17.47)	65.50 (14.29)	0.001
**Sex (Male, %)**	1059 (44.3)	33 (36.3)	438 (42.0)	398 (51.0)	190 (40.1)	<0.001
**SAPS-II (mean (SD))**	43.75 (14.66)	45.27 (14.82)	44.28 (14.23)	41.09 (14.92)	46.65 (14.45)	<0.001
**SOFA (mean (SD))**	4.20 (2.36)	4.01 (1.83)	4.14 (2.36)	4.03 (2.30)	4.62 (2.52)	<0.001
**LY-D1 (x10^9/L, mean (SD))**	1.12 (0.90)	2.49 (1.08)	0.83 (0.50)	1.70 (1.00)	0.51 (0.38)	<0.001
**LY-D2 (x10^9/L, mean (SD))**	1.04 (0.87)	1.67 (1.29)	0.79 (0.43)	1.80 (0.93)	0.34 (0.23)	<0.001
**LY-D3 (x10^9/L, mean (SD))**	1.00 (0.81)	0.88 (0.71)	0.79 (0.35)	1.90 (0.83)	0.30 (0.20)	<0.001
**LY-D4 (x10^9/L, mean (SD))**	1.05 (0.75)	0.47 (0.27)	0.91 (0.34)	1.82 (0.75)	0.32 (0.17)	<0.001
**HR (bpm, mean (SD))**	91.00 (17.48)	93.53 (17.32)	90.60 (17.34)	90.14 (17.07)	92.83 (18.33)	0.023
**T (℃, mean (SD))**	36.99 (0.61)	37.01 (0.58)	36.99 (0.63)	36.99 (0.58)	36.98 (0.61)	0.971
**RR (/min, mean (SD))**	20.98 (4.31)	21.07 (4.18)	21.00 (4.35)	20.97 (4.34)	20.93 (4.19)	0.991
**OI (mmHg. median [IQR])**	175.00 [110.00, 260.36]	200.00 [116.00, 301.00]	172.00 [108.75, 262.38]	174.50 [115.00, 252.38]	166.67 [100.00, 257.50]	0.462
**Lactate (median [IQR])**	2.30 [1.40, 4.50]	2.65 [1.72, 6.05]	2.20 [1.40, 4.30]	2.30 [1.50, 4.40]	2.70 [1.50, 4.90]	0.048
**Length of ICU stay (days, median [IQR])**	5.22 [3.17, 9.88]	5.11 [3.33, 8.62]	5.19 [3.15, 9.80]	5.18 [3.23, 9.74]	5.73 [3.03, 10.69]	0.810
**Duration of vasopressor (hours, median [IQR])**	65.00 [29.75, 149.25]	68.00 [37.00, 142.00]	67.00 [30.00, 152.00]	59.00 [30.00, 131.00]	76.50 [27.00, 163.50]	0.552
**Duration of mechanical ventilation (hours, median [IQR])**	68.50 [34.00, 146.25]	68.00 [35.00, 133.00]	74.00 [34.00, 151.00]	61.00 [30.00, 132.00]	85.00 [40.50, 160.00]	0.007
**28-Day Mortality (%)**	613 (25.7)	35 (38.5)	263 (25.2)	165 (21.2)	150 (31.6)	<0.001

Abbreviations: SD, standard deviation, IQR, interquartile range, SAPS II, Simplified Acute Physiology Score; SOFA, Sequential Organ Failure Assessment; HR, heart rate; T, temperature; RR, respiratory rate; OI, oxygenation index; LY, lymphocyte count; D1–D4, 1–4 days after intensive care unit admission.

Table S3. Comparison of clinical characteristics among the phenotypes of patients with sepsis in the prospective cohort.

Characteristic	Overall	α	β	γ	δ	*p*
n	1056	241	535	225	55	
Age (mean (SD))	60.0 (16.6)	63.8 (15.1)	57.1 (16.8)	62.5 (16.3)	62.6 (17.6)	<0.001
Sex (Male, %)	688 (65.2)	156 (64.7)	358 (66.9)	144 (64.0)	30 (54.5)	0.307
APACHE-II (mean (SD))	19.8 (7.5)	21.3 (7.5)	18.6 (6.9)	20.2 (7.8)	24.4 (7.6)	<0.001
SOFA (mean (SD))	6.9 (3.0)	7.0 (2.8)	7.0 (2.9)	6.6 (3.3)	6.7 (3.6)	0.353
HR (mean (SD))	102.1 (20.6)	102.8 (21.0)	100.7 (19.8)	103.4 (21.3)	107.5 (21.8)	0.062
T (mean (SD))	36.7 (1.0)	36.6 (1.0)	36.7 (0.9)	36.6 (1.0)	36.8 (1.0)	0.729
RR (mean (SD))	16.1 (7.7)	15.9 (6.7)	16.1 (8.5)	15.6 (6.6)	18.8 (7.9)	0.079
OI (mmHg, median [IQR])	211.9 [151.3, 281.2]	202.4 [144.6, 266.0]	219.3 [156.2, 288.9]	209.0 [152.5, 282.9]	179.5 [130.9, 262.9]	0.040
Lactate (mmol/L, median [IQR])	2.6 [1.7, 5.0]	3.0 [1.8, 6.0]	2.5 [1.6, 5.0]	2.4 [1.6, 4.1]	2.7 [1.8, 5.2]	0.020
BLC (mean (SD))	143.6 (150.1)	102.0 (88.5)	197.1 (180.2)	89.1 (72.3)	30.6 (31.3)	<0.001
TLC (mean (SD))	610.7 (394.0)	416.2 (199.4)	844.9 (389.2)	370.5 (203.5)	172.3 (197.8)	<0.001
CD4+TLC (mean (SD))	362.4 (246.4)	251.1 (140.0)	498.4 (251.0)	224.3 (133.2)	95.5 (110.5)	<0.001
CD8+TLC (mean (SD))	217.0 (182.9)	146.6 (109.8)	301.0 (202.7)	129.8 (97.8)	67.4 (88.6)	<0.001
CD4/CD8 (mean (SD))	2.3 (2.1)	2.4 (2.1)	2.2 (1.7)	2.5 (2.4)	2.4 (3.9)	0.426
NK-LC (mean (SD))	89.5 (89.3)	70.3 (71.4)	115.9 (102.1)	62.3 (56.7)	28.8 (38.3)	<0.001
Length of ICU stay (days, median [IQR])	7.2 [4.8, 16.8]	9.6 [4.8, 19.2]	7.2 [4.8, 14.4]	7.2 [2.4, 14.4]	9.6 [7.2, 18.0]	<0.001
Duration of vasopressor (hours, median [IQR])	112.3 [51.0, 261.6]	139.8 [64.9, 337.7]	105.0 [46.7, 237.1]	99.0 [45.2, 240.2]	165.9 [85.0, 333.1]	<0.001
Duration of mechanical ventilation (hours, median [IQR])	118.2 [55.9, 277.5]	148.2 [64.7, 364.4]	103.9 [48.0, 238.5]	113.8 [39.7, 266.1]	218.9 [130.8, 356.7]	<0.001
Infection source (%)						
CNS	2 (0.2)	0 (0.0)	2 (0.4)	0 (0.0)	0 (0.0)	0.008
SST	26 (2.5)	3 (1.2)	13 (2.4)	10 (4.4)	0 (0.0)	

Characteristic	Overall	α	β	γ	δ	*p*
**Valves**	57 (5.4)	10 (4.1)	40 (7.5)	6 (2.7)	1 (1.8)	
**Lung**	753 (71.3)	181 (75.1)	375 (70.1)	147 (65.3)	50 (90.9)	
**Intra-abdominal**	167 (15.8)	35 (14.5)	77 (14.4)	52 (23.1)	3 (5.5)	
**UTI**	3 (0.3)	1 (0.4)	1 (0.2)	0 (0.0)	1 (1.8)	
**Others**	13 (1.2)	3 (1.2)	8 (1.5)	2 (0.9)	0 (0.0)	
**Pleural**	19 (1.8)	5 (2.1)	11 (2.1)	3 (1.3)	0 (0.0)	
**Bloodstream**	15 (1.4)	3 (1.2)	8 (1.5)	4 (1.8)	0 (0.0)	
**Mediastinum**	1 (0.1)	0 (0.0)	0 (0.0)	1 (0.4)	0 (0.0)	
**28-Day Mortality (%)**	173 (16.4)	55 (22.8)	62 (11.6)	35 (15.6)	21 (38.2)	<0.001

Abbreviations: SD, standard deviation, IQR, interquartile range, APACHE-II, Acute Physiology and Chronic Health Evaluation II; SOFA, Sequential Organ Failure Assessment; HR, heart rate; T, temperature; RR, respiratory rate; OI, oxygenation index; BLC, B-lymphocyte count; TLC, T-lymphocyte count; NK, natural killer; CNS, central nervous system; SST, skin, and soft tissue; UTI, urinary tract infection.

Table S4. Clinical characteristics among patients in the prospective cohort categorized by 28-day mortality.

Characteristic	Overall	No	Yes	*p*
n	1056	883	173	
Age (mean (SD))	60.0 (16.6)	59.4 (16.6)	63.5 (16.2)	0.003
Sex (Male, %)	688 (65.2)	579 (65.6)	109 (63.0)	0.575
APA (mean (SD))	19.8 (7.5)	19.1 (7.0)	23.9 (8.5)	<0.001
SOFA (mean (SD))	6.9 (3.0)	6.9 (3.0)	6.8 (2.9)	0.772
HR (mean (SD))	102.1 (20.6)	101.1 (19.8)	107.3 (23.5)	<0.001
T (mean (SD))	36.7 (1.0)	36.6 (0.9)	36.9 (1.0)	<0.001
RR (mean (SD))	16.1 (7.7)	15.8 (7.8)	17.1 (7.4)	0.054
PFO_2 (mmHg, median [IQR])	211.9 [151.3, 281.2]	214.0 [156.2, 284.9]	181.7 [138.2, 265.4]	0.002
Lactate (mmol/L, median [IQR])	2.6 [1.7, 5.0]	2.6 [1.6, 5.0]	2.5 [1.8, 4.6]	0.800
BLC (mean (SD))	143.6 (150.1)	148.8 (154.9)	117.0 (119.1)	0.012
TLC (mean (SD))	610.7 (394.0)	630.9 (389.2)	506.0 (403.4)	<0.001
CD4+TLC (mean (SD))	362.4 (246.4)	371.7 (240.2)	314.3 (272.2)	0.005
CD8+TLC (mean (SD))	217.0 (182.9)	226.0 (183.3)	170.8 (174.0)	<0.001
CD4/CD8 (mean (SD))	2.3 (2.1)	2.3 (2.0)	2.6 (2.5)	0.037
NK-LC (mean (SD))	89.5 (89.3)	94.5 (90.0)	64.0 (81.4)	<0.001
Length of ICU stay (days, median [IQR])	0.3 [0.2, 0.7]	0.3 [0.2, 0.6]	0.5 [0.3, 0.9]	<0.001
Duration of vasopressor (hours, median [IQR])	112.3 [51.0, 261.6]	94.0 [45.2, 217.5]	261.7 [107.6, 468.7]	<0.001
Duration of mechanical ventilation (hours, median [IQR])	118.2 [55.9, 277.5]	104.5 [41.5, 230.2]	275.6 [136.1, 499.2]	<0.001
Infection source (%)				
CNS	2 (0.2)	2 (0.2)	0 (0.0)	<0.001
SST	26 (2.5)	23 (2.6)	3 (1.7)	
Valves	57 (5.4)	55 (6.2)	2 (1.2)	
Lung	753 (71.3)	599 (67.8)	154 (89.0)	
Intra-abdominal	167 (15.8)	155 (17.6)	12 (6.9)	
UTI	3 (0.3)	2 (0.2)	1 (0.6)	
Others	13 (1.2)	13 (1.5)	0 (0.0)	
Pleural	19 (1.8)	19 (2.2)	0 (0.0)	

Characteristic	Overall	No	Yes	*p*
**Bloodstream**	15 (1.4)	14 (1.6)	1 (0.6)	
**Mediastinum**	1 (0.1)	1 (0.1)	0 (0.0)	
**72-hour Lymphocyte trajectory phenotype (%)**				
**α**	241 (22.8)	186 (21.1)	55 (31.8)	<0.001
**β**	535 (50.7)	473 (53.6)	62 (35.8)	
**γ**	225 (21.3)	190 (21.5)	35 (20.2)	
**δ**	55 (5.2)	34 (3.9)	21 (12.1)	

Abbreviations: APACHE-II, Acute Physiology and Chronic Health Evaluation II; SOFA, Sequential Organ Failure Assessment; HR, heart rate; T, temperature; RR, respiratory rate; OI, oxygenation index; BLC, B-lymphocyte count; TLC, T-lymphocyte count; NK, natural killer; CNS, central nervous system; SST, skin and soft tissue; UTI, urinary tract infection.

A.

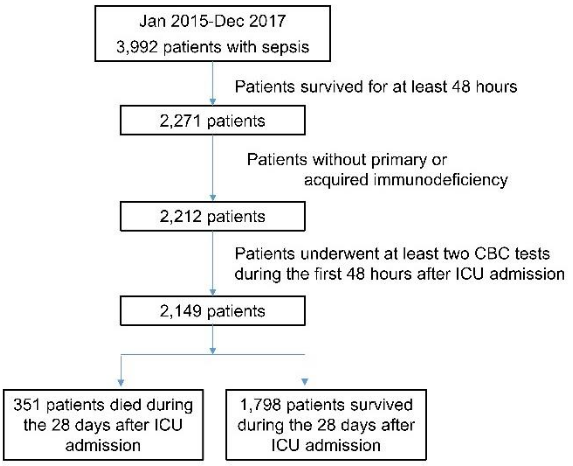

B.

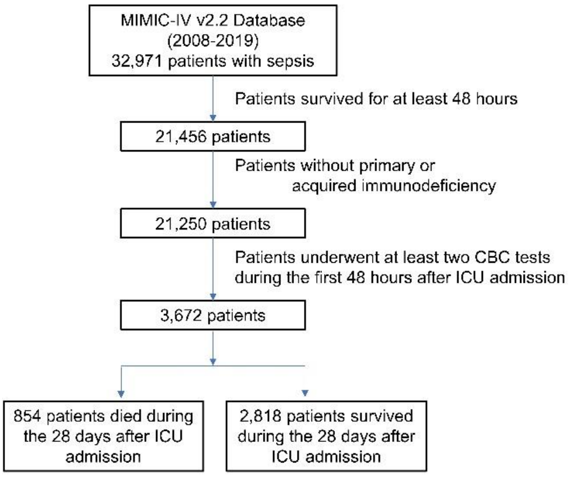

C.

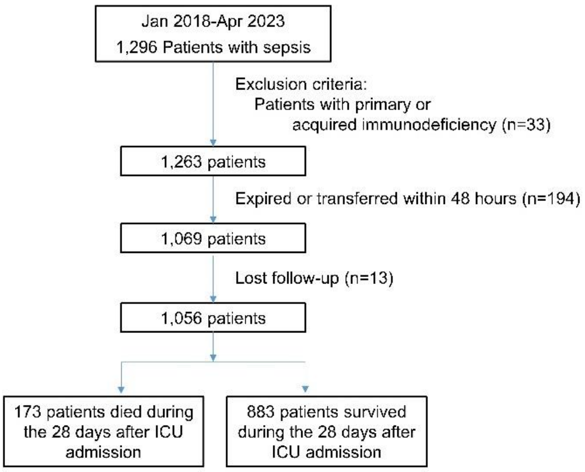

Figure S1. Flowcharts of the study protocol. A. Retrospective (PUMCH) cohort. B.

External validation (MIMIC-IV) cohort. C. Prospective (PUMCH) cohort. Abbreviations: MIMIC, Medical Information Mart for Intensive Care; PUMCH, Peking Union Medical College Hospital. ICU, Intensive Care Unit. CBC, Complete Blood Count.

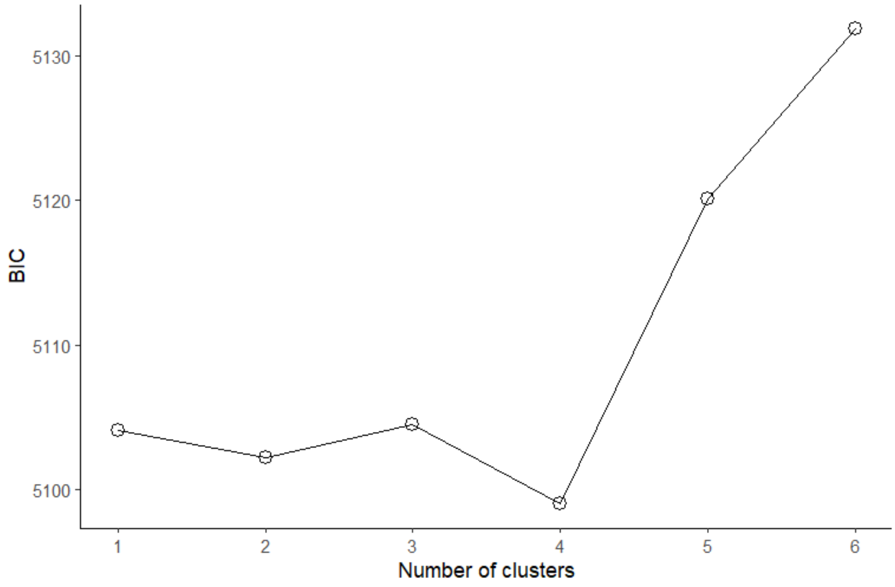

A.

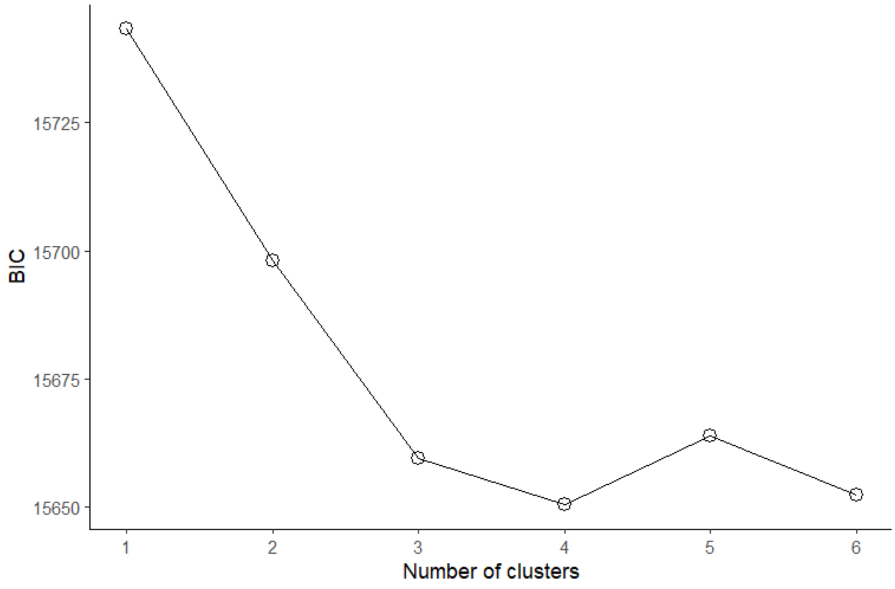

B.

Figure S2. Bayesian information criterion scores for various cluster number. The x-axis represents the number of clusters, while the y-axis represents the Bayesian information criterion value. A. Retrospective PUMCH cohort. B. External validation MIMIC-IV cohort. Abbreviations: MIMIC, Medical Information Mart for Intensive Care; PUMCH, Peking Union Medical College Hospital. BIC, Bayesian information criterion.

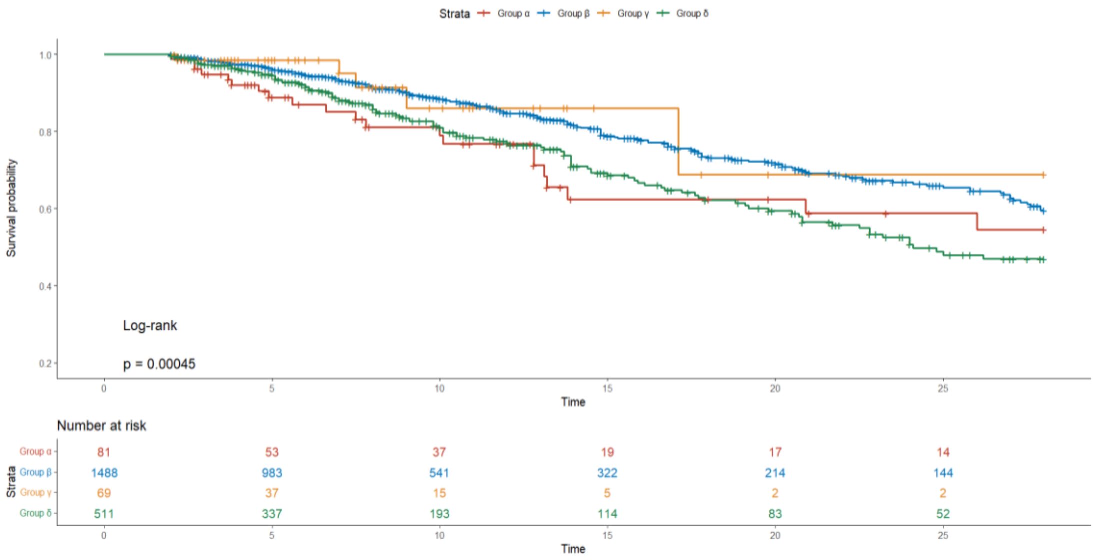

A.

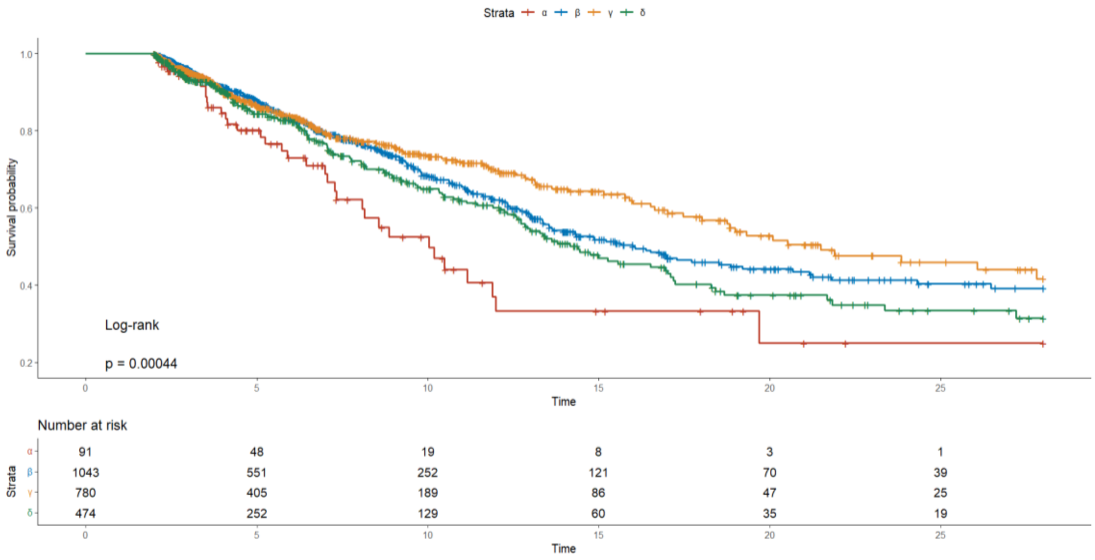

B.

Figure S3. Survival curves of 28-day mortality by phenotype. A. Retrospective (PUMCH) cohort. B, External validation (MIMIC) cohort. The *P* value (log-rank test) for the comparison among the survival curves of the four phenotypes at ICU discharge is shown. Abbreviations: MIMIC, Medical Information Mart for Intensive Care; PUMCH, Peking Union Medical College Hospital. ICU, Intensive Care Unit.

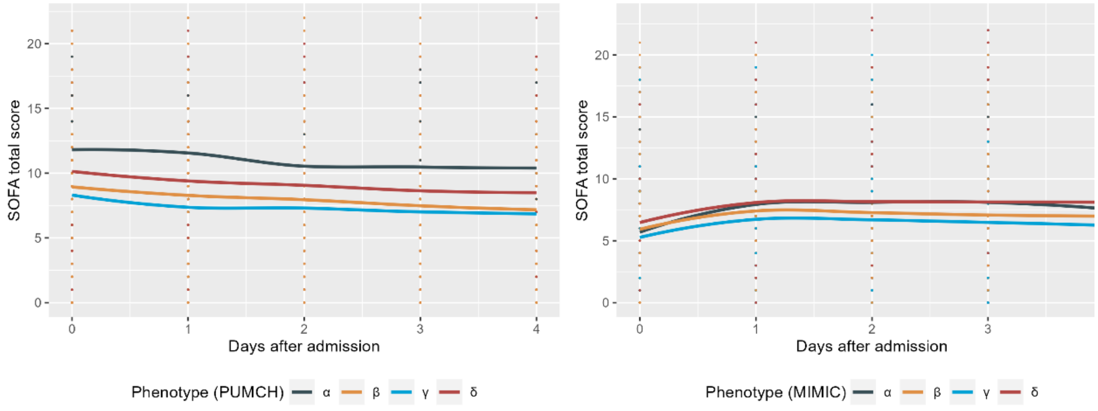
SOFA total score
Days after admission
Phenotype (PUMCH) α β γ δ
Phenotype (MIMIC) α β γ δ

A

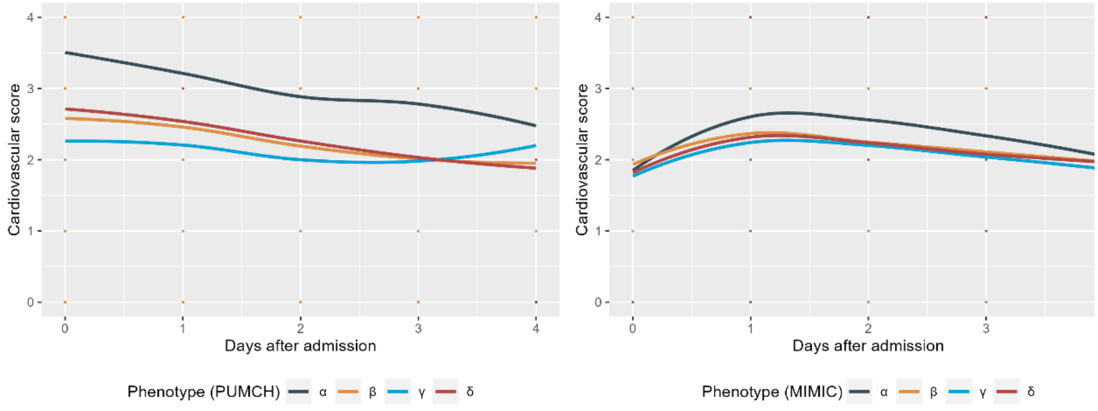
Cardiovascular score
Days after admission
Phenotype (PUMCH) α β γ δ
Phenotype (MIMIC) α β γ δ

B

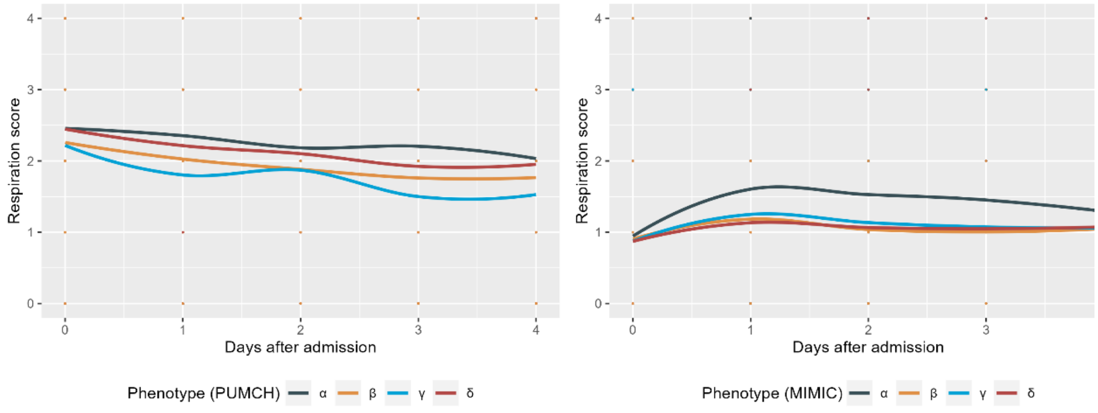
Respiration score
Days after admission
Phenotype (PUMCH) α β γ δ
Phenotype (MIMIC) α β γ δ

C

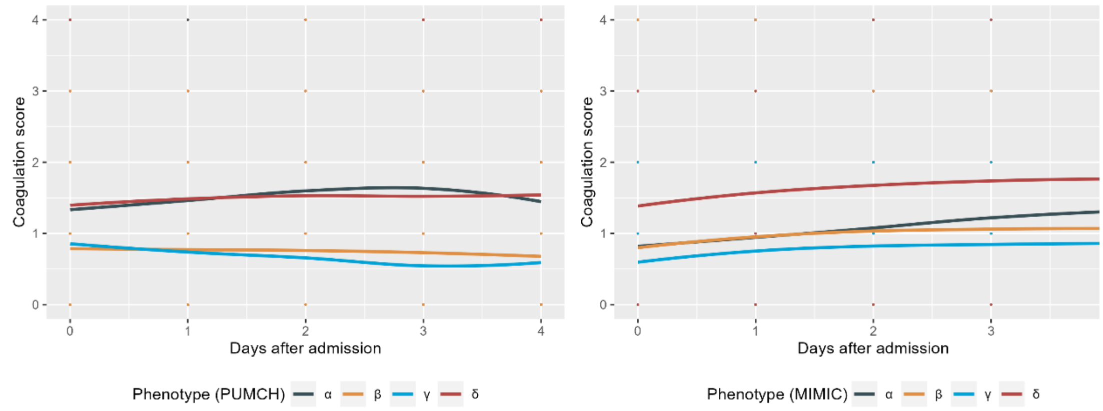
Coagulation score
Days after admission
Phenotype (PUMCH) α β γ δ
Phenotype (MIMIC) α β γ δ

D

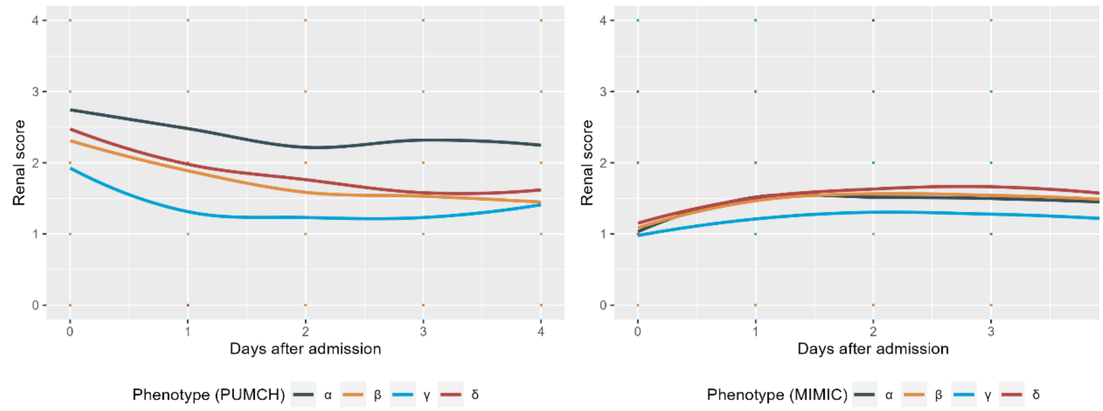
Renal score
Days after admission
Phenotype (PUMCH) α β γ δ
Phenotype (MIMIC) α β γ δ

E

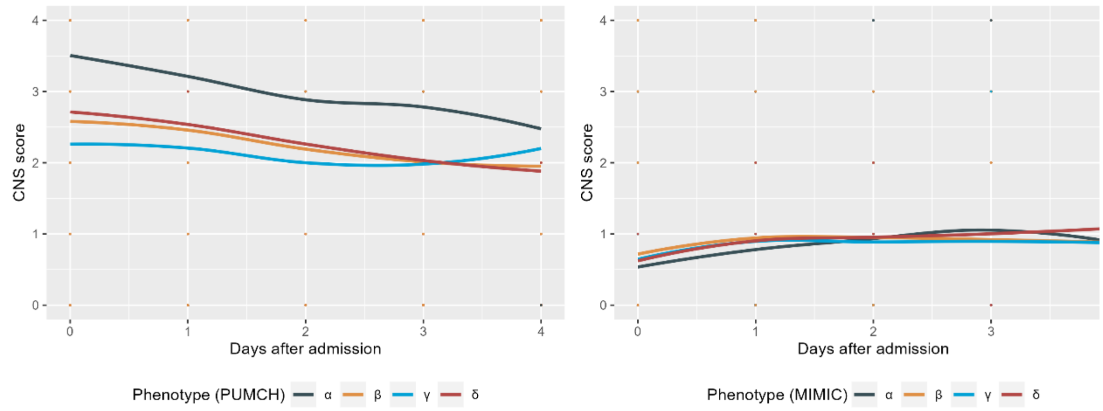
CNS score
Days after admission
Phenotype (PUMCH) α β γ δ
Phenotype (MIMIC) α β γ δ

F

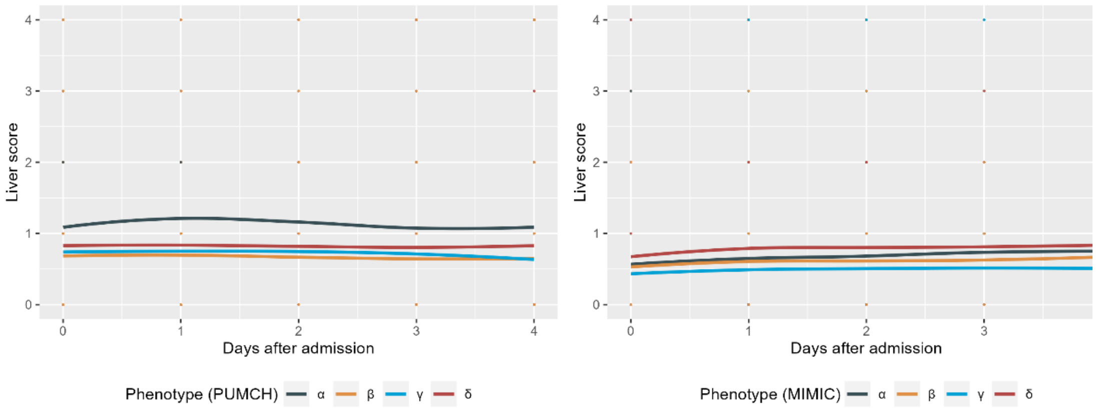

G

Figure S4. Longitudinal trajectory of the SOFA sub-score for each lymphocyte trajectory phenotype in the retrospective and external validation cohorts. The total SOFA score trajectory of each of the PUMCH cohort (A, left) and MIMIC-IV cohort (A, right) is illustrated in A, while the SOFA sub-score trajectory is represented in B–G. The organ systems are indicated on the y-axis. Abbreviations: MIMIC, Medical Information Mart for Intensive Care; PUMCH, Peking Union Medical College Hospital. SOFA, Sequential Organ Failure Assessment.

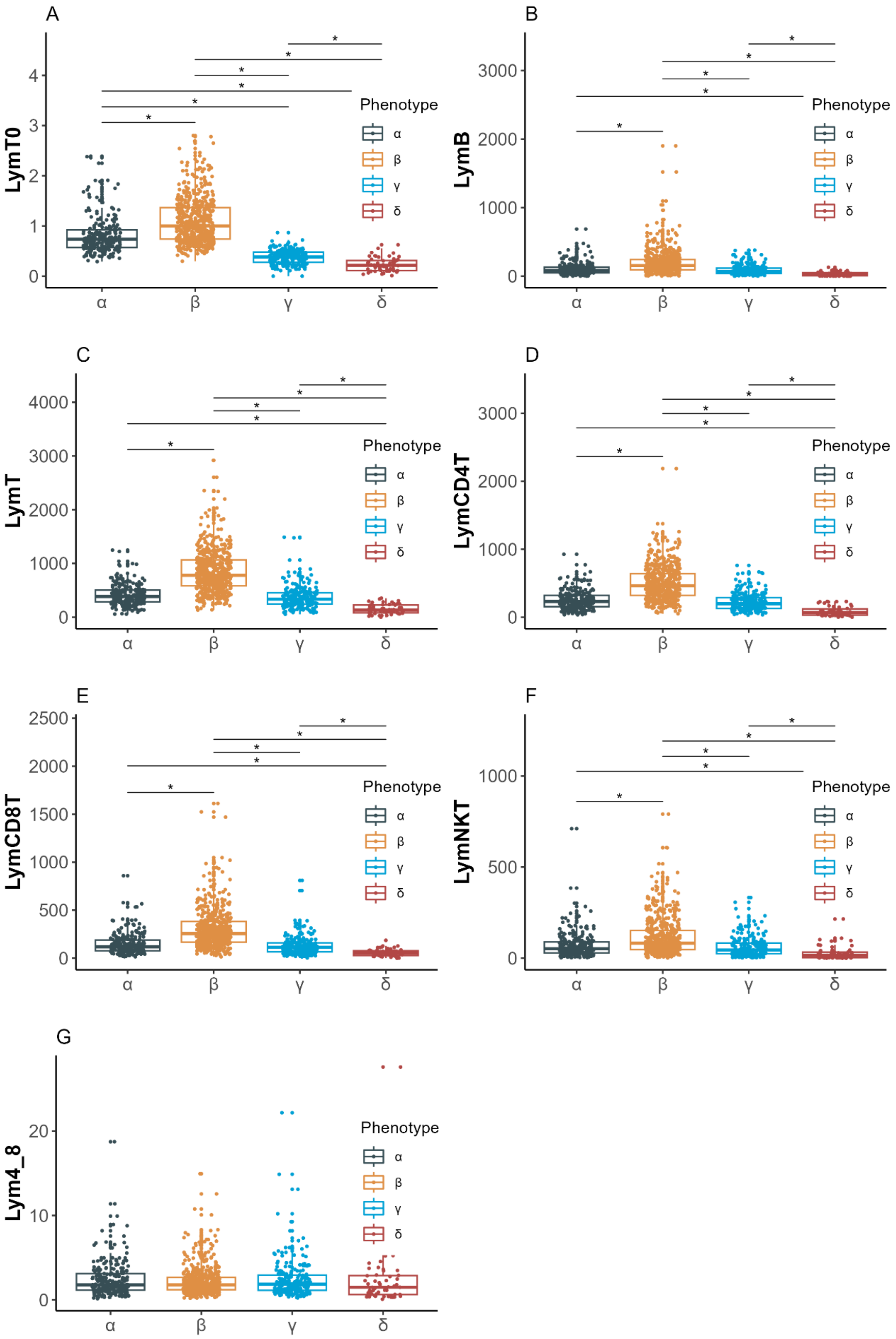

Figure S5. Comparison of lymphocyte subset counts among the four lymphocyte trajectory phenotypes in the prospective cohort. A–G represent the different

lymphocyte subsets (y-axis). LymT0, total lymphocyte count at intensive care unit admission; LymB, B-lymphocyte count; LymT, T-lymphocyte count; LymCD4T, $CD4^+$ T-lymphocyte count; LymCD8T, $CD8^+$ T-lymphocyte count; LymNK, natural killer lymphocyte count; Lym4_8, ratio of $CD4^+$ to $CD8^+$ T lymphocyte counts. The asterisk denotes statistical significance ($P < .005$).
